# Visual processing and collective motion-related decision-making in desert locusts

**DOI:** 10.1098/rspb.2022.1862

**Published:** 2023-01-25

**Authors:** Itay Bleichman, Pratibha Yadav, Amir Ayali

**Affiliations:** ^1^ School of Zoology, Tel Aviv University, 6997801 Israel; ^2^ Sagol School of Neuroscience, Tel Aviv University, 6997801 Israel

**Keywords:** *Schistocerca gregaria*, cognition, discrimination, visual processing, swarming

## Abstract

Collectively moving groups of animals rely on the decision-making of locally interacting individuals in order to maintain swarm cohesion. However, the complex and noisy visual environment poses a major challenge to the extraction and processing of relevant information. We addressed this challenge by studying swarming-related decision-making in desert locust last-instar nymphs. Controlled visual stimuli, in the form of random dot kinematograms, were presented to tethered locust nymphs in a trackball set-up, while monitoring movement trajectory and walking parameters. In a complementary set of experiments, the neurophysiological basis of the observed behavioural responses was explored. Our results suggest that locusts use filtering and discrimination upon encountering multiple stimuli simultaneously. Specifically, we show that locusts are sensitive to differences in speed at the individual conspecific level, and to movement coherence at the group level, and may use these to filter out non-relevant stimuli. The locusts also discriminate and assign different weights to different stimuli, with an observed interactive effect of stimulus size, relative abundance and motion direction. Our findings provide insights into the cognitive abilities of locusts in the domain of decision-making and visual-based collective motion, and support locusts as a model for investigating sensory-motor integration and motion-related decision-making in the intricate swarm environment.

## 1. Introduction

A fundamental aspect of all instances of collective motion is that of individual repeated decision-making [1–3]. This, in turn, is both driven by and relies on local interactions among the constituent agents, requiring each agent to obtain information about its surrounding social environment [4]. The consequent formation and maintenance of this distinctive form of synchronized movement is understood to be beneficial to the participating individuals [5–7].

**Figure 1.**
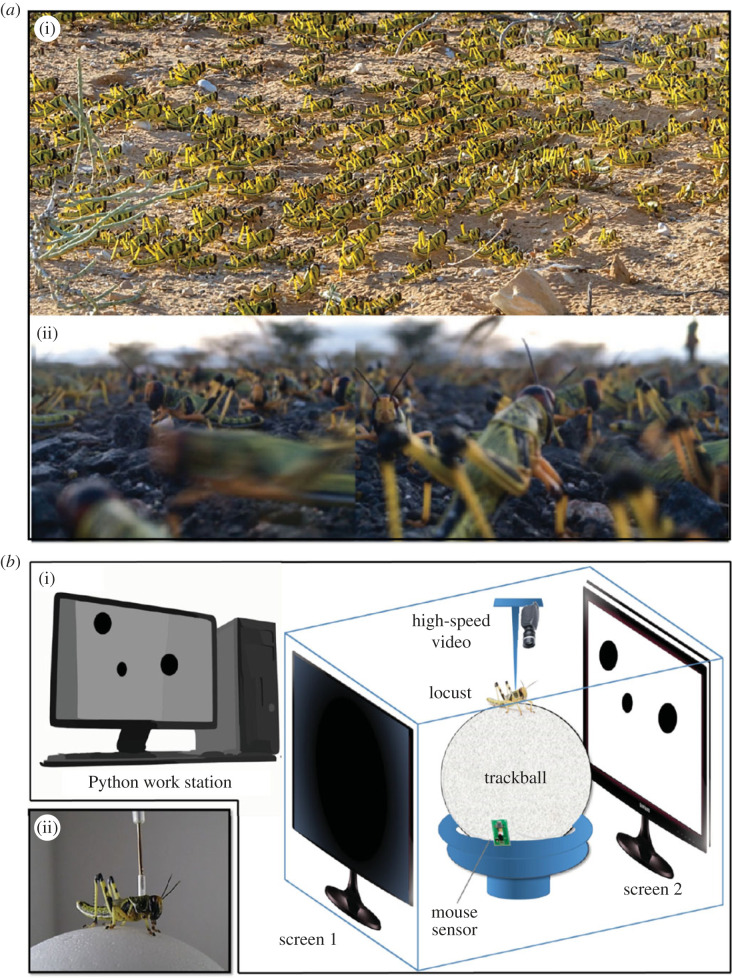
(*a*) Swarming desert locust nymphs. View from above (*a*)(i) (photo taken in Israel's Negev desert, 2013 by Gil Wizen), and a composite image from the ‘point of view’ of a locust (*a*)(ii) (photos taken in Kenya, 2020 by Inga Petelski). (*b*) Experimental set-up: complete set-up (*b*)(i) and individual tethered locust (*b*)(ii). An individual locust was tethered, with a fixed heading, over an airflow-suspended trackball. Random dot kinematograms were presented on two parallel LCD screens. High-speed video camera and a mouse sensor were used for behavioural tracking (their relative positions are only for illustration purposes).

A quintessential example of the above process is displayed by the desert locust, *Schistocerca gregaria* (Acrididae). When in the gregarious phase, they collectively move in huge dense marching swarms ([8,9]; figure 1*a*). Locust swarming is commonly accepted as heavily relying on visual perception [10]: each individual locust, with limited visibility amidst an unpredictable terrain, and an intricate, continuously changing social environment, must engage in repeated and dynamic decision-making to avoid getting derailed, while at the same time sustaining the collective motion. This can be translated into a two-layer process: the continuous extraction of the (unknown) state of the social surroundings from the input received by the sensory system (i.e. the eyes) and sensory-motor integration to facilitate the appropriate motor response. Different approaches, ranging from mathematical modelling to studying synchronization in small groups of locusts in laboratory settings, have been employed in the study of swarming behaviour in the desert locust [11–14]. However, our understanding of swarm formation and maintenance is still far from complete, partly owing to a lack of answers to some fundamental questions regarding decision-making at the individual level.

The swift extraction and processing of relevant information from a changing, complex sensory environment presents a critical challenge [15], especially in the noisy and cluttered visual surroundings of a locust swarm. Insects may adopt a range of strategies to increase the efficiency of information perception and processing by reducing the information load [16]. Such strategies include filtering relevant stimuli [17], categorizing the targets [18] and generalizing visual patterns [19]. Filtering relevant visual stimuli, for example by employing a ‘matched filter’ in the visual modality, can reduce the amount of information that needs to be processed [16,20]. In dragonflies and hoverflies, for example, small target detectors are specifically tuned to objects that constitute only a 1–3° angle of the visual field [21,22]. The filtering may occur at different levels of stimuli processing and vary with the ecological relevance of the stimuli. The insect's nervous system can then channel its resources into performing essential computations, even if complex, in order to extract the task-relevant visual information at low energetic cost [23]. In the case of the desert locust, we hypothesize that, during collective motion-related visual processing, the locust identifies and extracts relevant stimuli—swarming-related visual cues—from the overall visual scenery, based on a subset of visual features, enabling swift and appropriate decision-making. It is possible that a matched filter for walking speed is used to recognize marching conspecifics; while filtering based on the coherence of the moving group, as inferred from a subset of the swarm, might be used to estimate the overall direction of the swarm.

An additional difficulty imposed on information gathering can arise from the presence of multiple relevant competing inputs [24–26]. In this case, reducing the information load can also be achieved through selective attention—the ability to focus on one type of preferred stimulus while ignoring other perceivable ones [16,27]. There is accumulating evidence for attentional processes in insects, involving various sensory modalities [27–29]. Specifically for the visual system, the much related key capability to discriminate among different stimuli based on shape, colour and pattern orientation has been observed in honeybees and bumblebees [30–33], as well as in fruit flies, which show anticipatory behaviour consistent with selective attention to the tracked visual stimulus [34,35].

Desert locusts exhibit a characteristic pause-and-go motion, with pause duration correlated with a high probability of turning to change direction [36]. We can thus refer to the locust collective motion as comprising a series of repeated decisions taken by the individuals in the group [13]. Additionally, the decision-making process itself can be considered as a problem of vector selection, including a choice between continued standing or initiating walking, and a choice of direction. Observed variations in the fraction of time spent walking, and particularly in pause duration and the subsequent change in direction, in response to different visual stimuli, can thus offer valuable insights into the locust decision-making process.

We have previously shown that a specific motion-sensitive descending interneuron (DIN, one of many behaviourally relevant DINs (e.g. [37]), the descending contralateral movement detector (DCMD), conveys information relevant to the locust response to small, slow moving objects (such as other marching locusts [13] and see also [38,39]). Furthermore, this pathway was shown to demonstrate density-dependent phase-related differences [13,40], manifested in gregarious locusts being better suited than solitarious ones to the repeated decision-making, and thereby facilitating and coordinating the marching behaviour of the swarm. Monitoring the DCMD response to various swarming-related visual stimuli may offer some insights into the neural mechanisms behind the decision-making process under focus in this study.

Here we explored swarming-related decision-making at the behavioural level in *S. gregaria* nymphs, by analysing different aspects of the individual locust's walking behaviour. These served in our investigation of the role of visual feature recognition and discrimination as possible underlying mechanisms in decision-making. A complementary preliminary electrophysiological study of the processing of visual-motion inputs, relevant to the dynamic interactions between the individuals in a marching swarm, has lent further support to our hypotheses.

## 2. Methods

A more detailed description of the methods is provided in the electronic supplementary material.

### (a) Animals

All experiments were carried out using V^th^-instar larvae of *S. gregaria*, taken from our high-density, gregarious-phase locust laboratory-colony at the School of Zoology, Tel Aviv University (rearing conditions were described in [12]).

### (b) The experimental set-up

Individual locusts were tethered in a fixed (forward) head direction, via a 1 cm long clear vinyl tube attached to their pronotum with epoxy resin, in a natural-like typical walking posture, above an airflow-suspended Styrofoam trackball, illuminated from above with LED lights. The ball was decorated with an irregular black over white pattern in order to facilitate the tracking of its movement. Two parallel LCD screens, 30 cm apart, were positioned one on either side of the locust, allowing the presentation of controlled visual stimuli, while carefully monitoring the locust's behavioural responses and movements of the ball by a high-speed video camera (figure 1*b*). Experiments started after 1 h of acclimation of the locust to the tether. In two sets of behavioural experiments, the locusts' responses were monitored using FicTrac [41], a computer-vision tracking software that determines the angular position of the ball for each frame. In an additional set of experiments, an optical mouse sensor was further used to record the movement of the ball. The behavioural set-up was complemented by a corresponding electrophysiological set-up, enabling the recording of the neural responses of the locust DCMD interneurons to (similar) controlled visual stimuli (see Electrophysiology section below).

### (c) The visual stimuli

Visual stimuli, designed using the Python programming language version 3.9 (Python Software Foundation) and PsychoPy (an open-source software package; [42]), were presented in the form of random dot kinematograms (RDKs) of black dots on a white background, at 100% contrast. We chose RDK following previous reports of using such stimuli for testing multiple target processing, and specifically motion perception [43]. Unless stated otherwise, the RDK on each computer screen comprised forty, 1.2 cm diameter dots, each dot corresponding to a subtended visual angle of 4.58° on the insect's eye (within the known size of the locusts). Each visual stimulus was presented for 60 s. Dots were scattered equally on each of the two computer screens, and in random positions. In order to maintain a constant dot number on each of the screens, a dot disappearing at the edge of the screen was replaced by a new one appearing on the same screen at a random position.

We first presented the control stimuli: (i) blank (white screen) and (ii) still dots on a white screen. Next, we conducted a set of different behavioural experiments to investigate the tethered locust's response to the following different tentative features of swarming-related visual stimuli.

#### (i) Direction of motion

The RDK comprised fully coherent, 5 cm s^−1^ moving dots, simulating a coherently moving locust swarm. Three types of stimuli were used, each with a different direction of motion: (i) both screens showing dots aligned with the direction of the tethered locust's heading; (ii) both screens showing dots in a direction 180° to the tethered locust's heading; and (iii) one screen showing aligned dots and the other with dots moving in the opposite direction.

#### (ii) Motion speed

Tethered locusts were presented with dots moving with 100% coherence on both screens, aligned with the tethered locust's heading, and at graded speeds. The tested motion speeds were 1, 3, 5, 10 and 15 cm s^−1^, which cover a marching locust's speed range, as measured previously [12].

#### (iii) Coherence level

Tethered locusts were presented with dots moving on both screens at a motion speed of 5 cm s^−1^ and graded coherence levels: a fraction of the dots moved in alignment with the locust's heading while the remaining dots each moved in a random direction. Coherence levels tested were 0%, meaning all of the dots were moving in different random directions, 10% (i.e. 10% of the dots aligned with the locust heading and the remaining 90% moving in random directions), 25%, 50% and 100% (all dots aligned with the locust's heading direction).

#### (iv) Competing stimuli

A fourth experiment was conducted to investigate situations of competing stimuli, i.e. decision-making in the presence of conflict. Four types of stimuli were used: (i) two-thirds of the dots on each screen moved in a direction aligned with the tethered locust's heading, while the remaining one-third moved in the opposite direction; (ii) two-thirds of the dots on each screen moved in a direction 180° to the tethered locust's heading, while the remaining one-third moved in a direction aligned with its heading; (iii) the same as (i), but the one-third of the dots moving in the opposite direction to the two-thirds were double the size of the latter (2.4 cm diameter); and (iv) same as (ii) but the one-third of the dots moving in the opposite direction to the two-thirds were double the size of the latter. While the first two stimuli types present a more quantitative conflict, in stimuli (iii) and (iv) a size conflict, mimicking proximity differences, was also introduced.

### (d) Behavioural analysis

The rotation angle, the difference between two angular positions of the trackball in subsequent frames, was used to analyse the locust motion parameters. A motion threshold was determined based on the extent of the rotation angle. A locust was considered to be moving if the threshold was crossed for at least 10 consecutive frames. Pausing was determined if the same threshold was not crossed for at least 20 consecutive frames. Based on these indices, we calculated the fraction of time spent walking (walking fraction) and the average pause duration. A sideways motion threshold (positive for left side motions and negative for right side motions) was determined based on the direction of the trackball rotation. A locust was considered to be moving sideways if this threshold was crossed for at least 10 consecutive frames, and the total time spent walking sideways was calculated. The coordinate positions from the optical mouse sensor were used to determine the walking parameters by calculating the walking distance, using the Cartesian formula.

### (e) Electrophysiology

Dissection and electrophysiological procedures followed Ariel *et al*. [13]. Briefly, after CO_2_ induced anaesthesia, the legs of the nymphs were removed and a silver hook electrode was positioned around the ventral neck connectives for extracellular recording of DCMD activity. The locusts were positioned above a plastic platform in the same position and posture as on the airflow-trackball. The experiments were performed using RDKs with graded speeds and coherence levels similar to the behavioural experiments, and also using graded dot sizes of 0.4, 0.8, 1.2, 1.4, 2.2, 5.5 and 6.8 cm diameter. Each stimulus was presented for 20 s, with an inter-trial period of 1 min. The DCMD action potential times, number and frequency were analysed.

## 3. Results

### (a) The locusts respond to swarming-related visual stimuli with a preference to maintain their heading

**Figure 2.**
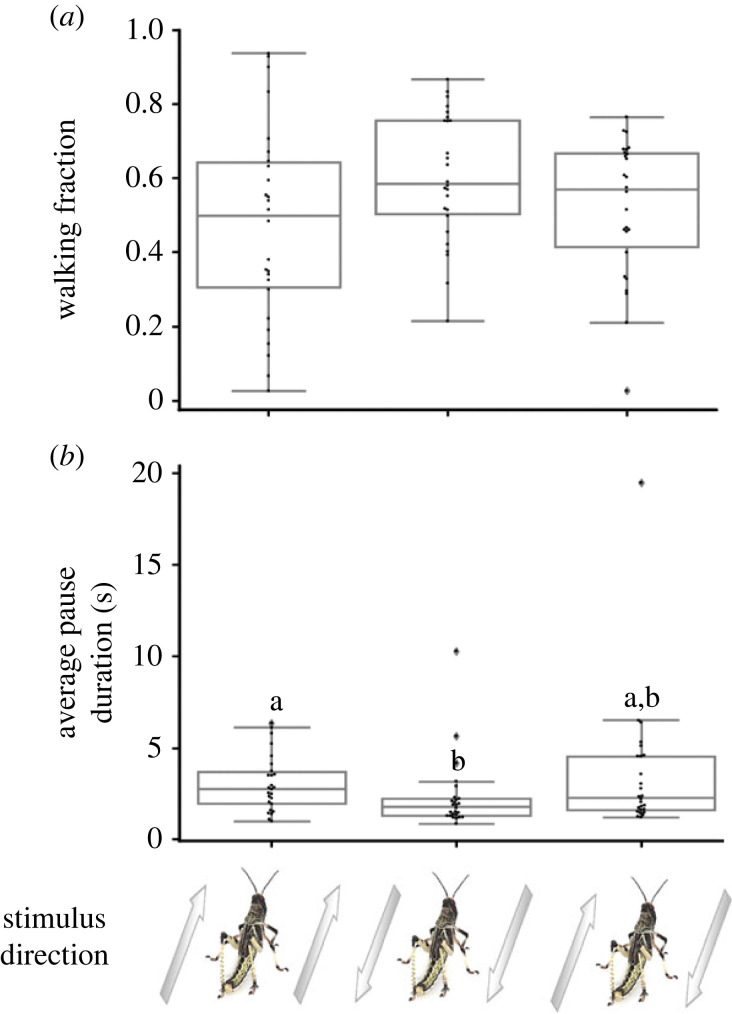
Locust response to swarming-related visual stimuli. Walking fraction (*a*) and average pause duration (*b*) in response to different motion directions of the stimuli. Arrows represent direction of motion relative to locust heading (aligned with locust heading, opposite to locust heading and one of the two screens aligned while the other is in the opposite direction). Each point represents data from a single locust (*n* = 26) Grey lines denote the median. Boxes show the interquartile range (25th to 75th percentiles). Whiskers include points up to 1.5 times the interquartile range. (*a*) No significant difference between different stimuli. (*b*) Significant difference is present between boxes with different letters.

**Figure 3.**
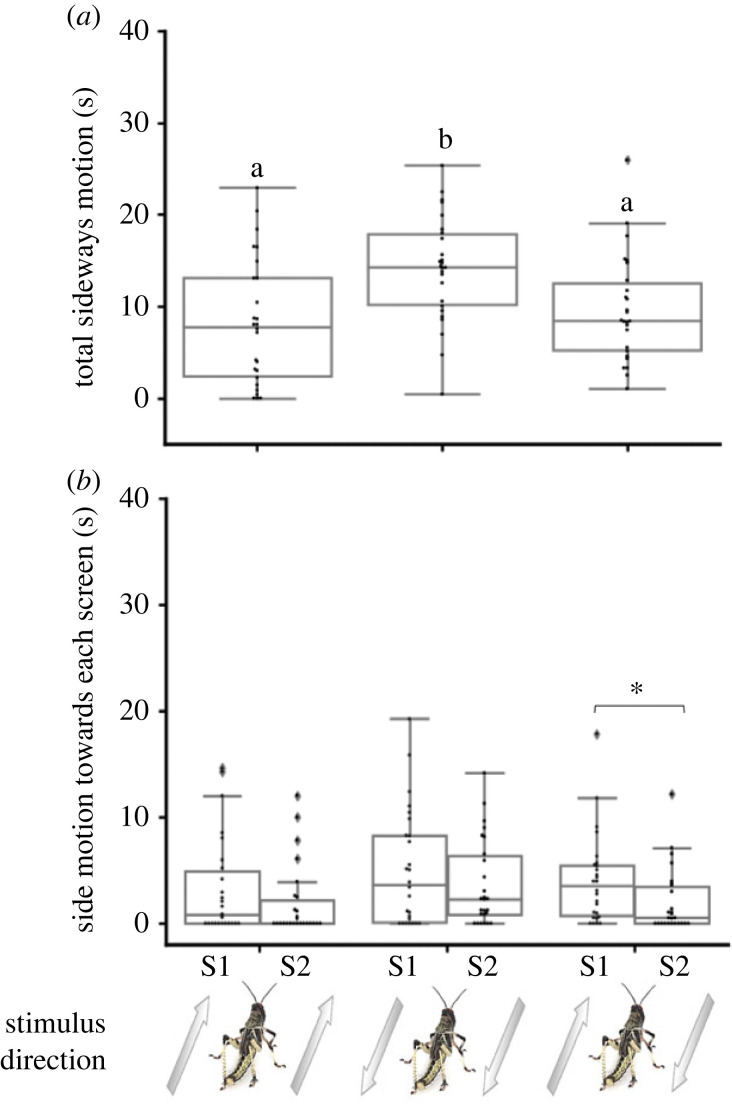
Locust response to swarming-related visual stimuli—side motion analysis. Total sideways motion time in seconds (*a*), and side motion time towards each specific side (S1 or S2) (*b*) in response to different motion directions of the stimuli. Arrows represent direction of motion relative to locust heading (aligned with locust heading, opposite to locust heading and one of the two screens aligned while the other is in the opposite direction). Each point represents data from a single locust (*n* = 26). Grey lines denote the median. Boxes show the interquartile range (25th to 75th percentiles). Whiskers include points up to 1.5 times the interquartile range. (*a*) Significant difference is present between boxes with different letters. (*b*) Significant difference marked with asterisk (*) (*p* < 0.05). In the first two cases S1 and S2 are interchangeable (same stimulus), in the last case one side is aligned and the other in the opposite direction, screens were intermittently switched between the left and right.

Locusts tethered in our set-up exhibited the typical pause-and-go motion pattern even in the absence of moving visual stimuli (electronic supplementary material, figure S1; see also [10,34]). Similar walking kinematics (manifested in pause duration and walking fraction) were measured in response to the white background only and to the background with motionless dots (electronic supplementary material, figure S2; *n* = 26, Wilcoxon matched-pairs signed rank test, *p* = 0.4742 for walking fraction and *p* = 0.3533 for pause duration). When testing the effect of moving stimuli, the response of the locusts comprised several clear time-dependent features (electronic supplementary material, figure S3). Specifically, when the direction of the stimulus motion on one or both screens was opposite to the tethered locust's head direction, the demonstrated behavioural response was not consistent throughout the stimuli, but became exhausted prior to termination of the stimuli. This time-limited response was probably owing to the open-loop nature of our experiments, i.e. to the fact that the locust's response did not induce any (expected) directional change in the incoming visual inputs. Consequently, we limited our comparative analysis of the different visual stimuli-induced behavioural responses to the first 40 s of each trial only. During this consistent and robust ‘responsive time-interval’, the locusts attempted to align themselves with the direction of motion of the dots, and/or to join the motion (figures 2 and 3).

When characterizing the behavioural response to stimuli with different motion directions, stimuli in which both screens showed dots moving in a direction 180° to the tethered locust's heading generated significantly decreased average pause duration compared to stimuli in which both screens showed dots aligned with the locust's heading (figure 2*b*; *n* = 26, Friedman test with Dunn's multiple comparisons test, *p* < 0.05) and significantly increased side motion compared to all other stimuli tested (figure 3*a*; *n* = 26, Friedman test, *p* < 0.001, Dunn's multiple comparisons test, *p* < 0.01 for all comparisons). Analysis of the single-sided motion i.e. only motion towards a specific side (towards one monitor or towards the other) revealed specific response to non-congruent stimuli. When each screen displayed a different direction of motion, one aligned with the locust's head direction and the other opposite to it, the locust side motion was significantly higher towards the screen displaying motion aligned with its heading compared to side motion towards the other screen (figure 3*b*; *n* = 26, Wilcoxon matched-pairs signed rank test, *p* < 0.05). It is important to note that in the non-congruent stimulus we were careful to switch between the left and right monitors intermittently. No such preference for side motion towards a specific screen was noted in the congruent stimuli, where both screens displayed the same direction of motion (figure 3*b; n* = 26, Wilcoxon matched-pairs signed rank test, *p* = 0.374 for stimuli aligned with locust heading and *p* = 0.426 for stimuli 180° to locust heading). Overall, these findings confirm the swarming-related nature of our controlled stimuli, i.e. the locusts clearly attempted to swarm alongside or to join the controlled visual stimuli presented in our experimental set-up, demonstrating a preference towards stimuli that were aligned with their initial heading.

### (b) Clear thresholds are demonstrated in the response to swarming-related visual stimuli at both the individual conspecific and the group level

**Figure 4.**
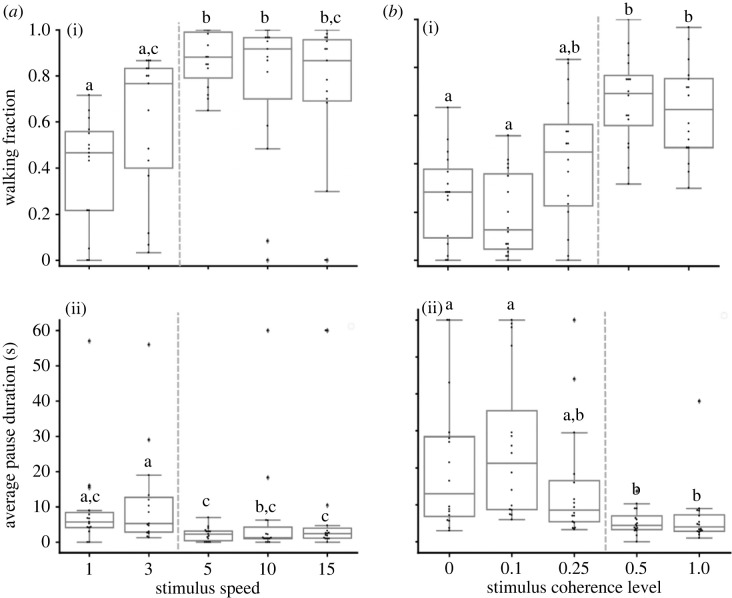
Behavioural thresholds in response to swarming-related visual stimuli. Average pause duration (*a*)(i) and walking fraction (*a*)(ii) in response to different stimulus motion speeds (*n* = 15). Average pause duration (*b*)(i) and (*b*)(ii) in response to different stimulus coherence levels (*n* = 16). Each point represents data from a single locust. Grey lines denote the median. Boxes show the interquartile range (25th to 75th percentiles). Whiskers include points up to 1.5 times the interquartile range. Points that are more than 1.5 times the interquartile range away from the bottom or top of the box are outliers. Different letters represent statistical differences. Grey dashed line indicates location of behavioural threshold.

The next feature investigated for a possible effect on the locust's behavioural kinematic parameters was motion speed. Comparing the generated behavioural response between the different stimuli, a clear motion speed dependence and a clear speed threshold were demonstrated: in response to stimuli with motion speed greater than 3 cm s^−1^, significantly higher walking fractions (figure 4*a*(i); *n* = 15, Kruskal–Wallis test, *p* < 0.0001) and shorter pause durations (figure 4*a*(ii); *n* = 15, Kruskal–Wallis test, *p* < 0.0001) were observed, compared to the response to dots moving at speeds below this threshold.

Maintaining the speed of all the moving dots above the demonstrated threshold, and changing the coherence level among the presented dots, revealed a second decision rule based on yet another threshold (figure 4*b*): in response to stimuli with coherence level above 25%, the locusts exhibited significantly larger walking fractions (figure 4*b*(i); *n* = 16, Kruskal–Wallis test, *p* < 0.0001) and significantly shorter pause durations (figure 4*b*(ii); *n* = 16, Kruskal–Wallis test, *p* < 0.0001) compared to their response to stimuli with coherence levels below this threshold. It is important to note that while both the speed and the coherence level are characteristic features of the visual inputs in a marching swarm, the former is a feature of each individual group member, contributing to the collective motion of the swarm; while the latter is a characteristic of the collective, or a group level trait, reflecting the common direction of motion.

### (c) The locust response to complex moving visual stimuli

**Figure 5.**
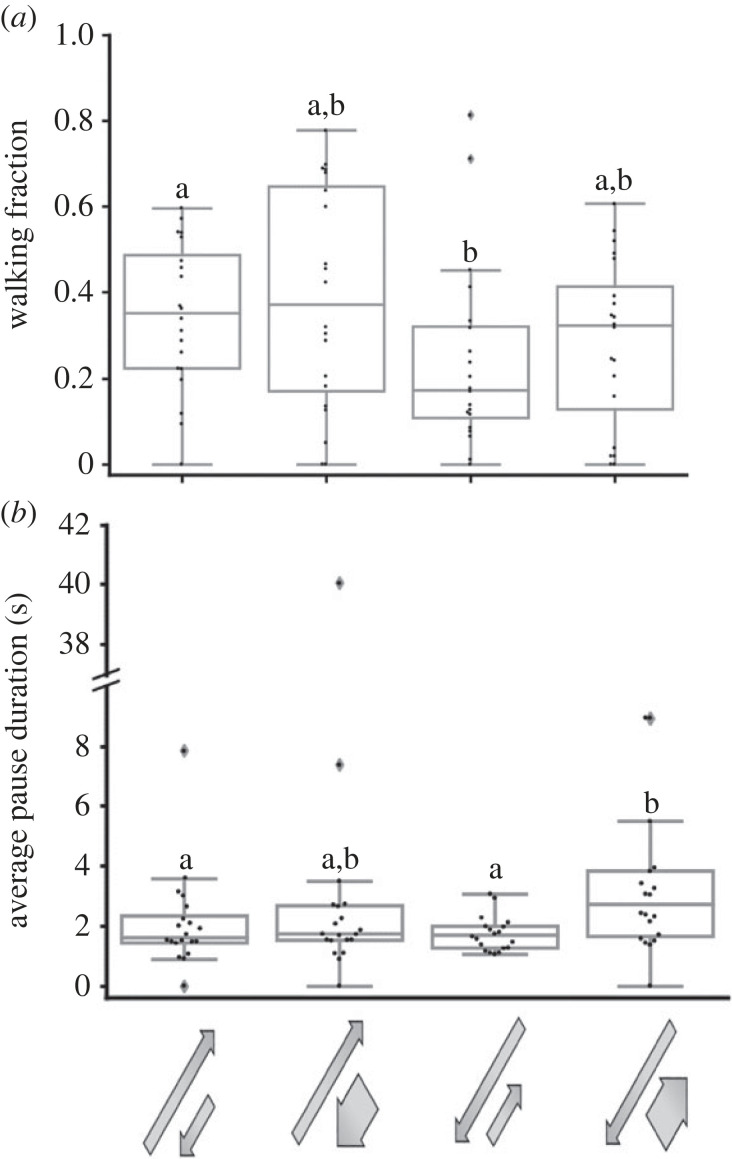
Size-direction-abundance interplay in response to complex visual stimuli. Walking fraction (*a*) and average pause duration (*b*). Arrows represent direction of motion. Arrow length represents relative abundance (long arrows = two-thirds of dots, short arrows = one-third of dots). Arrow width represents dot size (wide arrows—larger dots). Each point represents data from a single locust (*n* = 19) Grey lines denote the median. Boxes show the interquartile range (25th to 75th percentiles). Whiskers include points up to 1.5 times the interquartile range. Different letters represent statistical differences.

As noted, the visual environment within a locust swarm is an intricate and noisy one, leading us to investigate the locusts' response to complex and conflicting stimuli (figure 5). As described in the Methods section, four different stimuli were presented, confronting the locust with a mere quantitative (relative abundance) conflict, or a combined abundance and size conflict. Clear effects on the locust movement kinematic parameters were demonstrated only in stimuli in which the majority of dots were moving in a direction 180° to the tethered locust's heading. When all dots were equal in size and the majority of dots were moving in the opposite direction to that of the locust's heading, the locust's walking fraction significantly decreased compared to when the majority of equal-sized dots were aligned with the locust heading (figure 5*a*; *n* = 19, Friedman test, *p* < 0.05, Dunn's multiple comparisons test, *p* < 0.05). This was possibly owing to the conflict between the relative abundance (two-thirds of dots moving in the opposite direction) and the preferred motion direction (still present in the remaining one-third). Doubling the size of the dots in the one-third group, moving in the direction of the locust's heading partially restored walking fraction, but significantly increased pause duration compared to the two stimuli in which only equal-sized dots were used (figure 5*b*; *n* = 19, Friedman test, *p* < 0.01, Dunn's multiple comparisons test, *p* < 0.05). This specific complex visual stimulus required more intensive information processing by the locust, depicted by the longer pauses. Overall, these findings reveal intricate interactions between stimulus number, size and direction, which together affect the locust decision-making process.

### (d) Neurophysiological correlates to the responses to swarming-related visual cues

Further exploration of the sensory-motor processing of swarming-related visual cues was conducted through a series of neurophysiological experiments.

**Figure 6.**
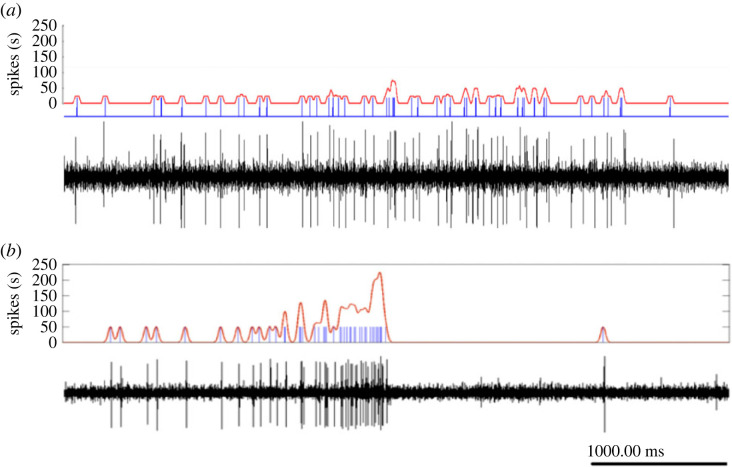
Typical response of the DCMD to swarming-related (*a*) and looming (*b*) stimuli. DCMD spike occurrence times (blue) were extracted from the extracellular recordings (black). Individual raster trials were then smoothed with a 20 ms Gaussian window and an evaluation of the instantaneous firing rate (red) was calculated. Recording (*a*) is the responses to swarming-related visual stimuli with motion speed of 5 cm s^−1^ and recording (*b*) is a response to looming stimulus (modified from Ariel *et al*. [13]).

Based on the behavioural observations, we expected our different visual stimuli to induce variable neuronal responses, depending on the stimuli motion speed, coherence level and dot size. This hypothesis was tested by studying the response of the DCMD interneuron, a key participant in a well-described motion-sensitive visual pathway [44,45], to similar types of stimuli as above. The DCMD has been mostly studied in the context of looming stimuli. Hence, it should be noted that the responses observed and monitored in our experiments differ from those of the typical looming response (figure 6). The DCMD average firing rate in response to control stimuli (white background and still dots) was similar to its spontaneous firing rate reported in previous studies (0.805 ± 0.273 and 0.707 ± 0.308 spike s^−1^ respectively) [13]. Manipulating the characteristics of swarming-related visual stimuli thus induced different responses.

#### (i) Motion speed

**Figure 7.**
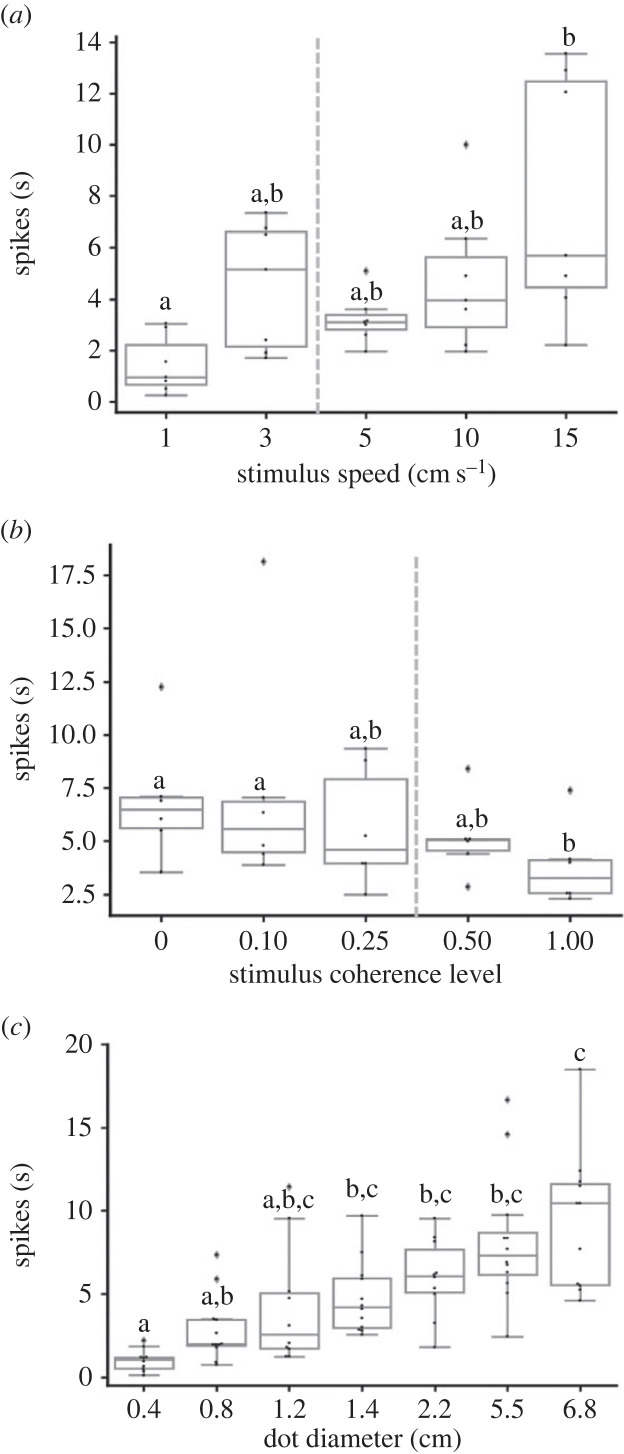
DCMD response to different motion speeds (*a*), coherence levels (*b*), and dot size (*c*) of swarming-related visual stimuli. Each point represents data from a single locust ((a) *n* = 7, (b) *n* = 6, and (c) *n* = 10). Grey lines denote the median. Boxes show the interquartile range (25th to 75th percentiles). Whiskers include points up to 1.5 times the interquartile range. Different letters represent statistical differences. (*a*) and (*b*): grey dashed line indicates value of behavioural threshold.

We found a clear dependence of the DCMD firing rate on the speed of the moving dots. Comparing all the different motion speeds tested (1, 3, 5, 10 and 15 cm s^−1^), a significant difference was seen between the slowest tested motion speed: 1 cm s^−1^ and the fastest one: 15 cm s^−1^ (figure 7*a*; *n* = 7, Friedman test, *p* < 0.001, Dunn's multiple comparisons test, *p* < 0.001), comprising two extremes just outside the speed range of marching locusts [12]. The DCMD's responses to visual stimuli moving at intermediate speeds did not significantly differ from each other.

#### (ii) Coherence level

Low coherence levels elicited high DCMD firing rates, with the DCMD response declining with the increase in motion coherence level from 10% to 100% (figure 7*b*). DCMD firing rate with coherence levels of 0% or 10% was significantly higher compared to that in response to 100% coherent stimuli (figure 7*b*; *n* = 6, Friedman test, *p* < 0.01, Dunn's multiple comparisons test, *p* < 0.01).

#### (iii) Size effect

Testing the DCMD response to swarming-related moving stimuli comprising dots of different sizes, revealed a size-dependent firing rate: a significant difference was noted between dots with a diameter of 0.4 cm and those with a diameter of 2.2, 5.5 or 6.8 cm (figure 7*c*; *n* = 10, Kruskal–Wallis test, *p* < 0.0001, Dunn's multiple comparisons test, *p* < 0.05). A graded increase in firing rate was seen with the increase in size.

Overall, our neurophysiologic investigation revealed that the DCMD was sensitive to different speeds, coherence levels and sizes of swarming-related visual stimuli, in a similar though not identical manner to that revealed in our behavioural experiments.

## 4. Discussion

Sensory information has a crucial role in ecological decision-making [15]. In order to enable sensory processing to be swift and context-appropriate, organisms are required to identify and extract highly specific, behaviourally relevant, signals from their surroundings [46]. Different strategies for rapidly coping with a visually cluttered environment have been suggested in previous studies of different organisms engaged in vision-based collective motion [13,47–49]. Flocking birds were reported to consider visual information from a fixed number of influential neighbours (i.e. a topological range; [50]). Zebrafish rely on visual occupancy for direction choice [47] and use bout-like movements for conspecific recognition [51]. Collectively moving *Drosophila* larvae depend on the number of conspecifics and cues related to their unique visual kinematic for decision-making [52]. Beyond the principal role of motion sensitivity in maintaining synchrony during collective marching [11,12], only very limited knowledge is available regarding how locusts use visual-sensory cues for swarming-related decision-making amidst their highly challenging visual surroundings.

Our findings have identified specific characteristics of the behaviourally relevant visual inputs affecting decision-making in desert locust nymphs. Moreover, we show, to the best of our knowledge for the first time, that locusts can extract collective motion relevant information at both the individual conspecific level (i.e. speed) and the group level (coherence or common direction), possibly by means of filtering and discrimination. While filtering can reduce the information processing load at the very first stage by differentiating relevant from non-relevant stimuli and ignoring the latter, discrimination can aid the extraction of information from the relevant stimuli and subsequently facilitate critical decision-making.

Desert locust nymphs walk at an average speed of approximately 5 cm s^−1^ [12]. In response to coherent stimuli moving at non-zero speeds below 3 cm s^−1^, the tethered individuals exhibited longer pause durations and shorter walking fractions, possibly reflecting longer decision-making time owing to a mismatch between dot speed and the expected behaviourally relevant conspecifics' speed. Hence, the locusts employed filtering at the level of the characteristic of the individual. In natural settings, such a clear speed threshold may be exploited to recognize marching conspecifics, such that anything moving at a speed below the threshold is ignored. This behavioural threshold was discovered in the V^th^-larval instar nymphs (corresponding to walking speed at this stage). Since locust collective marching appears early on and is maintained throughout the different developmental (larval) stages [53], and as development is accompanied by a marked change in size as well as in walking speed, an interesting point for future research would be that of developmental plasticity within the observed speed threshold.

The relatively limited walking behaviour demonstrated in response to dots moving in a non-coherent fashion (a non-decisive state manifested by unusually long pause durations) could have resulted from a lack of appropriate relevant information at the level of the group movement pattern. Importantly, our findings indicate that the locusts perceive a complete absence of motion cues (i.e. still dots) differently to that of an absence of conclusive information in the motion cues (i.e. non-coherently moving dots). This could be reflected in gregarious locusts continuing to walk even when alone (albeit with altered kinematics [14], but tending to pause and wait when surrounded by conspecifics moving randomly, e.g. early morning at the initial organizing stages of a swarm in a natural setting [53,54]). This ability to extract the trajectory of the surrounding locusts seems to be a fundamental characteristic of gregarious-phase locusts, instrumental for the decision-making in enabling the collective motion of locust swarms.

In his review, Warrant [55] discusses different visually matched filters and their important role in the ecology of vision in insects. These include peripheral matched filters for sex (mate), for prey detection and pursuit, and for the physical environment (aspects of the physical terrain). Central visual matched filters include filters for the insect's own locomotion speed (‘fast’ or ‘slow’ eyes) and for navigation (the celestial pattern of polarized light). Our above findings suggest yet another possible matched filter that is crucial for locust swarming behaviour: this can be referred to as a ‘social environment’ matched filter, or maybe even filters, as we have demonstrated filtering at both the level of the motion of individual neighbouring conspecifics as well as of the surrounding group.

Relevant conflicting stimuli increase the difficulty imposed on information processing and decision-making. A conflict can derive from a contradiction in one feature (e.g. opposing motion directions), requiring a single-attribute decision, or from the complex interactions between several features (e.g. direction, abundance and size), becoming a multi-attribute choice problem [56]. Locusts presented with directionally contradicting but otherwise identical swarming-related visual stimuli, demonstrated a preference for stimuli with motion direction aligned with their own heading. This preference to join in the marching aligned to the locust's current heading is consistent with the observation that marching locusts in experimental ring-shaped arenas only rarely change direction [11,13]. When different parameters provide conflicting or inconsistent information, it becomes beneficial to assign a higher weight to one over the other. Ants, for example, rank several attributes when faced with a multi-feature problem [57], and the ranking is dynamic in relation to their current situation [58]. When presented with mixed-type stimuli, size affected the locust's behaviour only when in a specific relation to the direction of motion and relative abundance. The interplay between these three parameters significantly affected pause duration, which is the kinematic phase assumed to be dedicated to information processing and decision-making [13,36]. Size may act as a proxy for visual target distance, with nearest neighbours being larger. More attention dedicated to larger dots, although less abundant, indicates that immediate neighbours may influence the decision of an individual more strongly than more distant members of the swarm. This is also consistent with previous reports suggesting a limited functional radius of attention around an individual in a group [13,14].

The DCMD is one out of several currently known DINs that take part in conveying visual, motion-related information [59,60]. It has been widely researched for its characteristic response to looming visual stimuli, and its function in predator and collision avoidance manoeuvres [61–64]. Nevertheless, the motion-sensitive pathway in which it takes part is also capable of responding to different, complex types of visual-motion stimuli [39,64]. The DCMD was also shown to demonstrate activity changes with developmental stages [38]. Ariel *et al*. [13] explored the effect of swarming-related visual information on the locust decision-making, demonstrating DCMD's ability to convey information regarding looming and receding stimuli with locust marching-related characteristics, and a specific ‘tuning’ of the response habituation rate in swarming, gregarious-phase locusts compared to the non-swarming solitarious-phase ones. Here we add another layer, exploring the effect of different types of swarming-related visual stimuli, i.e. other stimuli that are prevalent in the visual field of a locust within a marching swarm. The DCMD in our experimental setting demonstrated consistent responses to visual (non-looming) motion stimuli, with a clear effect of changes in motion speed, coherence level and stimulus size. The sensitivity to changes in these specific stimulus features emphasizes their importance and supports their involvement in visual information processing and swarming-related decision-making.

As also noted by Ariel *et al*. [13], we do not imply that the DCMD is the key interneuron governing swarming-related decision-making, but, rather that it is a good model. This interneuron's response to swarming-related visual stimuli, to which locusts demonstrated clear behavioural responses, can provide information regarding the neurophysiological mechanisms underlying these behavioural responses and the related decision-making process.

In its simplest form, decision-making can be understood as the process of selecting between two alternatives. The flexibility of decisions is accepted as a trademark of higher cognition in organisms [65]. Given that cognitive performances and integral abilities are often assumed to be positively correlated with brain size across species, it is no surprise that the miniature brains of insects are believed to limit their computational power and cognitive abilities [66–69]. However, with mounting evidence in support of highly sophisticated behaviours in insects [70], this assumption currently holds little ground. Here, we have provided additional insights into insect cognition by investigating decision-making in a collectively marching insect species. We demonstrate that desert locusts use discrimination, mediated by selective attention, to extract relevant information from a complex, noisy, visual environment. The rules of decision-making (decision rules) in gregarious desert locusts seem to be a function of multiple interacting factors, with the ultimate goal of staying in synchronization with conspecifics. A differential weightage to parameters such as direction, number and size of the stimuli was also observed, with conflicting information increasing the difficulty imposed on the decision-making process.

To conclude, locusts use different mechanisms that enable them to meet the challenges presented by the overloaded and cluttered visual environment, and that supports the sensory perception and integration required for collective motion-related decision-making. These mechanisms constitute an instrumental aspect of their ability to synchronize with conspecifics and maintain the cohesion of the swarm and thus probably also exist in all animals demonstrating visual-based collective motion. Much further work is required, however, in order to uncover and describe the details of the sensory-motor integration (e.g. the role of feedback from the motor system), to fully elucidate the underlying neurophysiological mechanisms (e.g. additional key neuronal pathways) and to provide insights into related brain-level phenomena, such as the representation of conspecifics and their behaviour (speed, direction, etc.), the actual depiction or abstraction of the swarm as a whole, and more.

## Data Availability

Electronic supplementary material is available online on Figshare [71]. The data are provided online on Dryad Digital Repository: https://doi.org/10.5061/dryad.jdfn2z3dw [72].
